# Congenital intrahepatic portocaval shunts and hypoglycemia due to secondary hyperinsulinism: a case report and review of the literature

**DOI:** 10.1186/s13256-018-1881-y

**Published:** 2018-11-12

**Authors:** Alexander Weigert, Jeanette Bierwolf, Heiko Reutter, Ulrich Gembruch, Joachim Woelfle, Rainer Ganschow, Andreas Mueller

**Affiliations:** 1 Clinic of General Pediatrics, University Hospital Bonn, Bonn, Germany; 2 Department of Neonatology and Pediatric Intensive Care, University Hospital Bonn, Bonn, Germany; 3 Institute of Human Genetics, University of Bonn, Bonn, Germany; 4 Department of Obstetrics and Prenatal Medicine, University of Bonn, Bonn, Germany; 5 Pediatric Endocrinology Division, Children’s Hospital, University of Bonn, Bonn, Germany; 6 Universitätskliniken Bonn – Zentrum für Kinderheilkunde, Adenauerallee 119, 53113 Bonn, Germany

**Keywords:** Case report, Congenital portosystemic shunts (CPSS), Intrahepatic portocaval shunt, Hypoglycemia, Secondary hyperinsulinism

## Abstract

**Background:**

Congenital portosystemic shunts present with various associated complications, such as other congenital malformations, hyperammonemia, or hepatopulmonary syndrome. Few cases of associated hypoglycemia have been reported so far and our case, to the best of our knowledge, describes the most severe extent of hypoglycemia.

**Case presentation:**

We describe the case of a newborn Arab boy with two intrahepatic portosystemic shunts, resulting in severe and persistent hypoglycemia, due to which one of the shunts was closed by interventional radiology whereas the other shunt had already closed spontaneously.

**Conclusions:**

Because he showed elevated levels for insulin and prolonged high insulin levels in an oral glucose tolerance test, our case supports the theory that portocaval shunts cause a reduced hepatic insulin reduction due to the high blood volume bypassing the liver. This case provides further insights into glucose regulation mechanisms of the liver and we suggest a consistent screening for hypoglycemia in patients with congenital portosystemic shunts.

## Introduction

Congenital portosystemic shunts (CPSS) are rare congenital malformations of the portocaval vessel system (prevalence is 1 in 30,000) [1]. CPSS are associated with various complications mostly secondary in nature including hyperammonemia along with neurological restrictions, liver tumors, elevated conjugated bilirubin levels, hepatopulmonary syndrome [2], and additional congenital malformations; congenital heart disease represents the most frequent associated congenital malformation [1].

CPSS can be divided into intrahepatic and extrahepatic shunts based on their anatomy. Intrahepatic shunts have a higher rate of spontaneous closure [3]. In contrast, extrahepatic shunts almost never show spontaneous closure. Hence, surgical intervention or occlusions by interventional radiology techniques represent state-of-the-art treatment [1]. Closure of the shunt should be considered in all extrahepatic shunts and patients with intrahepatic shunts with signs of encephalopathy, hepatopulmonary syndrome, or portopulmonary hypertension [2].

Here we report a case of a newborn with two congenital intrahepatic portosystemic shunts with concomitant, severe persistent hypoglycemia due to secondary hyperinsulinism. We describe the clinical course and treatment and review the literature.

## Case presentation

Our patient is the fourth child of two non-consanguineous, healthy Arab parents. His elder siblings are healthy. Prenatal ultrasound showed two intrahepatic shunts originating from the left portal main branch and concomitant cardiomegaly, the ductus venosus was normally located and patent. After normal spontaneous delivery, the male newborn was admitted to our neonatal intensive care unit for further diagnostics and clinical monitoring.

The prenatal diagnosis of cardiomegaly was confirmed postnatally by echocardiography and a non-compaction cardiomyopathy was diagnosed. Since our patient had no signs of cardiac output failure and normal cardiac function, no therapy was necessary during the neonatal period.

**Fig. 1:**
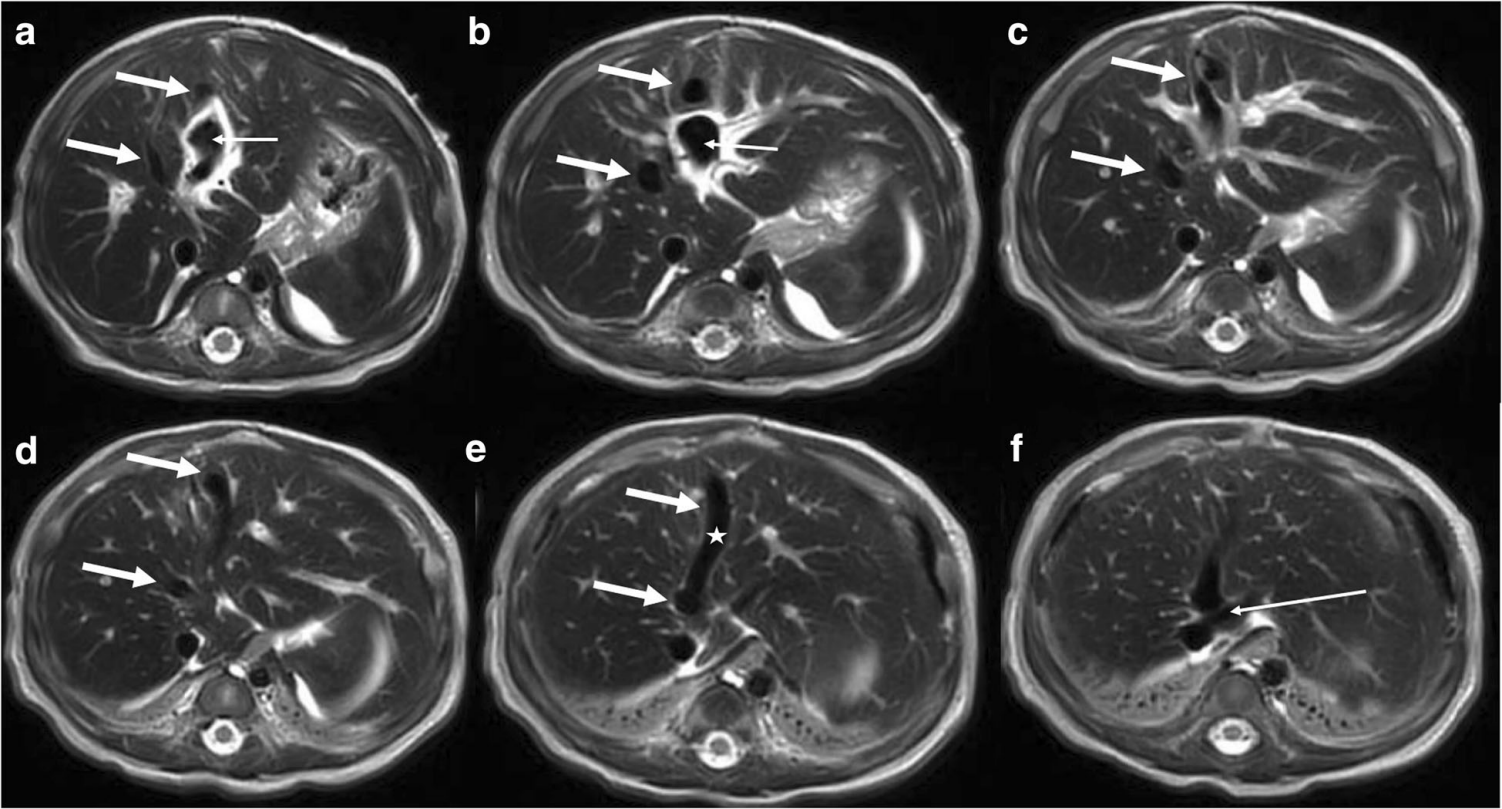
Series of abdominal axial magnetic resonance images showing the two intrahepatic shunts. **a**–**f** Caudal to cranial. The two shunts (marked by *thick white arrow*) originate from the recessus umbilicalis (marked by *thin white arrow* in a and b) of the left portal branch, draining into the dilated medial liver vein (*white star* in **e**) and then into the vena cava inferior (*white thin arrow* in **f**)

Postnatal ultrasound and magnetic resonance imaging (MRI) examinations confirmed the diagnosis of two intrahepatic shunts originating from the left portal main branch draining into the middle portal branch which appeared to be dilated (maximum 6 mm) (Fig. 1e). Blood serum levels showed only initially elevated levels of gamma-glutamyltransferase (gamma-GT), while alanine aminotransferase (ALT) and aspartate aminotransferase (AST) showed increased blood levels. The newborn developed hyperbilirubinemia which required 2 days of phototherapy. He also developed a clinically relevant neonatal cholestasis, which was treated with ursodeoxycholic acid, L-carnitine, and liposoluble vitamins (higher administration of these vitamins is required since the intestinal absorption of these vitamins require bile acids [4]). Due to a decreasing synthesis of coagulation factors he received one fresh-frozen plasma concentrate and regular, orally administered vitamin k substitutions. Ammonia was only slightly elevated and serum galactose, as an expression of shunt-capacity (galactose from milk bypassing the liver), was normal throughout the clinical course. From the second day of life, our patient presented with clinically relevant hypoglycemia (blood sugar below 45 mg/dl) which was treated with intravenously administered glucose substitutions. Efforts to regain a normoglycemic state by increased oral glucose substitution were unsuccessful. Even with an oral glucose uptake of 25 g per kilogram body weight per day episodes of hypoglycemia reoccurred. Increased oral glucose uptake was achieved both by adding glucose and starch to our patient’s nutrition. Starch (for example, glycoside), in contrast to glucose, is processed slower in newborns, leading to a prolonged elevation of blood sugar, without creating high blood sugar peaks [5]. Measured insulin in a hypoglycemic state showed elevated levels of insulin.

Due to these findings, an orally administered therapy with diazoxide was started to suppress insulin secretion. Simultaneously starch was added to the newborn’s milk feeding. As a result, blood sugar levels stabilized and periods of hypoglycemia were scarce, but still present. A scheme of oral glucose uptake and resulting hypoglycemia is presented in Table 1.

**Table 1:** Scheme of oral glucose uptake and resulting hypoglycemia

Age (days)	Glucose content of nutriment (g/kg per day)	Medication/dietary supplement	Range of hypoglycemia (mg/dl)
6	14.5	Maltodextrin	35–44
10	18.4	Maltodextrin FM85 5%	19–38
28	22	Maltodextrin 200 ml/kg Milk uptake	30–44
36	23.2	+ Diazoxide	28–42
80	17.5	Starch diazoxide	34–44
100	17.7	Starch diazoxide	41–44

**Fig. 2:**
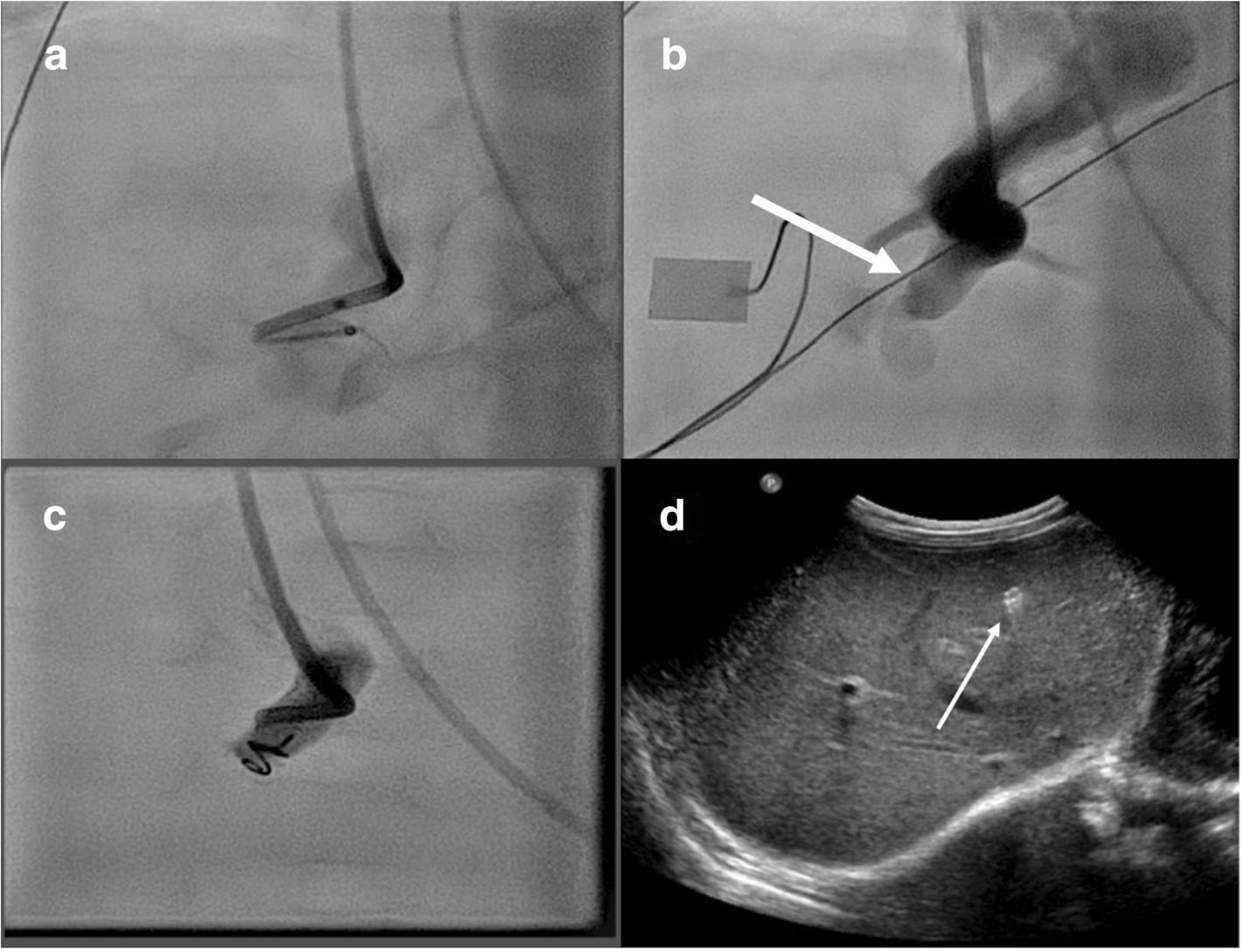
Closure of the remaining shunt. **a**–**b** The shunt (*white arrow*) during the intervention. **c** After the coil has been positioned. **d** Post-interventional control via ultrasound (coil marked by *white arrow*)

We decided to close the shunts via interventional radiology due to the recurrent episodes of hypoglycemia, insufficient production of coagulation factors, and the elevated levels of ALT and AST. When he was 2 months of age the first shunt was closed by coil and the other had already closed spontaneously (see Fig. 2). After the intervention, his blood sugar levels stabilized, as did the coagulation factors and ALT/AST levels began to decrease.

Our patient was discharged at 3 months of age. His parents were taught how to measure blood sugar levels and instructed to do so three times a day to make sure the therapy with diazoxide and Glycosade® (modified cornstarch)-enhanced mother’s milk was sufficient. The diazoxide therapy showed no side effects. Close follow-up examinations including an ultrasound of his liver were warranted because patients with congenital portocaval anastomosis have an increased risk for developing liver tumors in the first years of life [2].

### Literature review

We searched the literature (www.ncbi.nlm.nih.gov; April 30, 2017) for reports of hypoglycemia in patients with intrahepatic portocaval shunts and hypoglycemia due to secondary hyperinsulinism using the following search terms: “intrahepatic,” “portocaval,” “portosystemic,” “shunts,” “hypoglycemia,” and “hyperinsulinism.” The results are summarized in Table 2.

**Table 2:** Case reports of hypoglycemia in patients with intrahepatic portocaval shunts and hypoglycemia due to secondary hyperinsulinism

Author	Title	Shunt anatomy	Therapy
Bas *et al*., 2015 [9]	Premature pubarche, hyperinsulinemia and hypothyroxinemia: novel manifestations of congenital portosystemic shunts (Abernethy malformation) in children	Extrahepatic	Dietetic (rich in complex carbohydrates)
Duprey *et al.*, 1985 [7]	Glucose intolerance and post-stimulatory hypoglycemia secondary to a probably congenital intrahepatic portocaval anastomosis	Data not available	Data not available
Gouin *et al*., 1984 [6]	Congenital intrahepatic portocaval anastomosis: analysis of manifested glucose abnormalities	Data not available	Data not available
Senniappan *et al*., 2015 [10]	Postprandial hyperinsulinemic hypoglycemia secondary to a congenital portosystemic shunt	Extrahepatic	Diazoxide, surgical closure
Yoshii *et al*., 2017 [11]	Portosystemic shunt as a cause of congenital hyperinsulinemic hypoglycemia	Data not available	Data not available
Present case	Congenital intrahepatic portocaval shunts and hypoglycemia due to secondary hyperinsulinism: presentation of a new case and review of the literature	Intrahepatic	Interventional closure

## Discussion

Congenital portocaval shunts represent a rare malformation with a great variety of clinical symptoms, ranging from asymptomatic cases up to early death due to secondary complications such as hepatopulmonary syndrome or pulmonary hypertension. Nowadays, CPSS are often diagnosed prenatally due to improved ultrasound techniques; however, diagnosis is still occasionally made by chance based on biochemical abnormalities in routine screening procedures. A classification based on the anatomy (intrahepatic versus extrahepatic shunts) and especially decision making based on this classification has been questioned recently [2], because the same severe complications may affect both groups. On the other hand, this classification can be helpful, since intrahepatic and extrahepatic shunts have different chances of spontaneous closure [3].

Complications such as hypoglycemia due to hyperinsulinism as reported in our case seem to be rare [1, 6, 7] and, until now, never led to the decision of closing an intrahepatic shunt. Sokollik *et al.* provided a very feasible algorithm for the management and diagnosis of CPSS [2]. The authors proposed screening for CPSS via ultrasound if, for example, neonatal conjugated hyperbilirubinemia or hypergalactosemia is present. We propose to add “persistent hypoglycemia” to the reasons for screening for CPSS. When CPSS is diagnosed, Sokollik *et al*. suggested screening for complications such as encephalopathy, developmental delay, hepatopulmonary syndrome, or pulmonary hypertension to decide whether a closure of the shunt is necessary [2]. We propose, although it seems to be a rare complication, to add a consequent and regular screening for low blood sugar levels to screen for persisting hypoglycemia to these algorithms. This can be achieved either through regular measurement of blood glucose levels during the first 2 days of life or by using a continuous glucose monitoring system.

The episodes of hyperinsulinism with secondary hypoglycemia reported here might result from a reduced hepatic degradation of insulin due to the high blood volume bypassing the liver. A previous study on rats showed that portocaval shunts are associated with an increased storage of insulin in the pancreatic B cells and a reduced synthesis of secretory proteins for insulin, with normal serum levels for insulin [8]. The authors described these effects as a result of reduced insulin degradation in the liver [6]. This effect has also been described in a few other case reports [9, 10]. Bas *et al.* postulated an insufficient postprandial hepatic glucose uptake, resulting in early systemic hyperglycemia, leading to an exaggerated insulin secretion [9]. In addition, the insulin then bypasses the hepatic metabolism, causing late hypoglycemia due to the prolonged insulin effect [7].

Since our patient also presented with elevated levels for insulin and prolonged high insulin levels in an oral glucose tolerance test, our case supports the previous findings of Bani *et al*. [8], suggesting that the portocaval shunts cause a reduced hepatic insulin reduction. Surprisingly, in our patient, serum levels for ammonia or galactose were normal. One should expect hyperammonemia and/or hypergalactosemia due to the increased blood volume bypassing the liver and thus the hepatic metabolism. Why both hyperammonemia and hypergalactosemia were absent in our patient cannot be conclusively explained. This probably would require experimental animal model studies, which would be beyond the scope of this case report. It is worth noting that in other cases of CPSS with hypoglycemia due to hyperinsulinism, levels of ammonia were also normal [7, 8].

## Conclusions

The blood volume bypassing the liver does not only alter the hemodynamics of patients with CPSS it also, as our case shows, has a strong influence on metabolic homeostasis. Although it seems that hypoglycemia is a rare complication of CPSS, we suggest a persistent screening for hypoglycemia in those patients. If CPSS are diagnosed prenatally, then regular measurement of blood sugar levels during the first 2 days of life (similar to children of diabetic mothers) should be performed, either through regular blood glucose analysis or using continuous glucose monitoring.

## Abbreviations

ALT: Alanine aminotransferase
AST: Aspartate aminotransferase
CPSS: Congenital portosystemic shunts
Gamma-GT: Gamma-glutamyltransferase
MRI: Magnetic resonance imaging
